# Supplementary material for: A nickase Cas9 gene-drive system promotes super-Mendelian inheritance in Drosophila
Source: Cell Rep. Author manuscript; Available in PMC 2022 Jun 13. (PMC9190248; doi:10.1016/j.celrep.2022.110843)
Supplement: Supplemental figures [file NIHMS1810669-supplement-Supplemental_figures.pdf]

Cell Reports, Volume *39*

Supplemental information

A nickase Cas9 gene-drive system promotes super-Mendelian inheritance in *Drosophila*

Víctor López Del Amo, Sara Sanz Juste, and Valentino M. Gantz

Supplementary Information

Supplementary figure 1

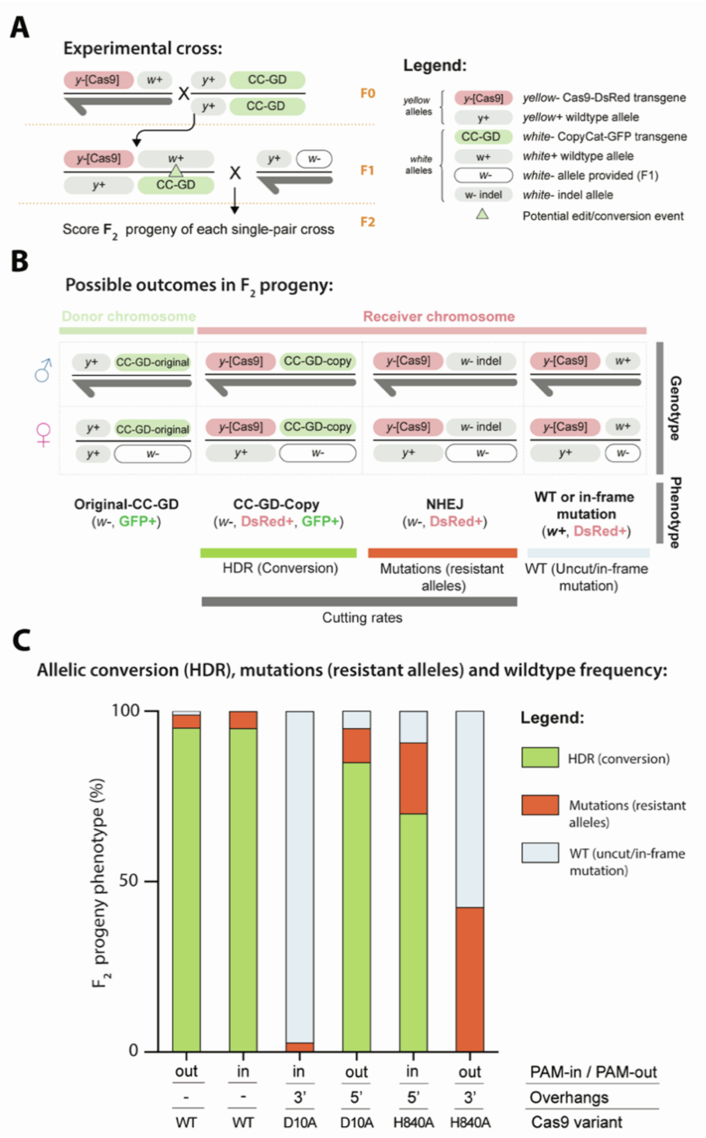

Fig S1 - Related to Fig.2 - Allelic conversion (HDR), mutations (resistant alleles) and wildtype (WT; uncut or in-frame mutations) rates produced by the regular Cas9 and nickase versions combined with our CopyCat elements. This analysis represents the outcomes produced by the wildtype Cas9 and nickase versions when combined with our *white* CopyCat experiments in **Figure. 2b,c**. **a.** Experimental cross with alleles present in our experimental design are depicted. *Yellow* and white genes are located 1.5 centimorgans in the X chromosome of *Drosophila*. The chromosome containing our Cas9 sources (inserted in *yellow*), and which (wildtype) *white* gene is targeted by the gene-drive element for conversion in F1 females, represents our receiver chromosome. Our CopyCat element (CC-GD) is inserted in *white*, representing our donor chromosome (chromosome of origin of the gene-drive element). **b.** The results were graphed according to three possible categories based on the phenotypic readouts: 1) Allelic conversion or HDR – alleles that were converted (presenting as DsRed+, GFP+ and white eye), 2) resistant allele events – alleles that were cut but were not converted (presenting as DsRed+, GFP- and white eye), and 3) wildtype – these individuals displayed wildtype eyes (red color or mosaic), DsRed+ and GFP-, suggesting that these alleles were not acted upon. **c.** The cutting efficiency ([conversion + NHEJ] / total) for wildtype Cas9 was ~100% when using both gene-drive CopyCat elements. Wildtype/uncut alleles were almost not detected, Cas9 combined with PAM-out showed only 1% uncut alleles while Cas9 PAM-in displayed 0% uncut alleles. Considering the conversion efficiency, both Cas9 situations showed 94-95% HDR rates. nD10A generating 5' overhangs produced 85% conversion, 10% mutations and 5% wildtype of the targeted alleles. nH840A generating 5' overhangs produced 70% conversion rates and 21% mutations, wildtype alleles represented 9% in this situation. nD10A and nH840A producing 3' overhangs did not trigger HDR. In this same condition where nickases generated 3' overhangs, nD10A only induced 3% cutting and 97% wildtype alleles. nH840A produced ~41% cutting and 59% wildtype/uncut alleles (see calculation on **Supplementary Data 1** and **Supplementary Data 2**).

Supplementary figure 2

(A) Large deletions (>50nt)

nD10A PAM-out

```
WT  :  CGATCGCCGCAG    GGCATCCAAGTATCGCCATCCGGGATGCGACTGCTCAATGGCCAAC    CTGTGGACGCCAA
       CGATC·······    ··············································    CTGTGGACGCCAA      (-53bp)
       CGATCGCCGCAG    GGC···········································    ··········CAA      (-53bp)
```

nH840A PAM-in

```
WT  :  TTGGCCGTGATGGGCAGTTCCGG    TGCCGGAAAGACGACCCTGCTGAATGCCCTTGCCTTTCGATCGCCGCAG    GGCATCCAAGTATCGCCATCCGGGATG
       TTGGCCGTGATGGGCAGTTCCGG    ·················································    ··CATCCAAGTATCGCCATCCGGGATG    (-51bp)
       TTGGCCGTGATGGGCAGTTCC··    ·················································    ···ATCCAAGTATCGCCATCCGGGATG    (-54bp)
       TTGGCCGTGATGGGCAGTTCC··    ·················································    ·······AAGTATCGCCATCCGGGATG    (-58bp)
       TTGGCCGTGATGGGCAGTT····    ·················································    ·····CCAAGTATCGCCATCCGGGATG    (-59bp)
       TTGGCCGTGATGGGCAGT·····    ·················································    ···········ATCGCCATCCGGGATG    (-65bp)(X4)
       TTG····················    ·················································    ···························    (-96bp)
       TTGG···················    ·············ACCCTGCTGAATGCCCTTGCCTTTCGATCGCCGCAG    GG······················ATG    (-54bp)
```

Cas9 PAM-out

```
WT  :  CGATCGCCGCAG    GGCATCCAAGTATCGCCATCCGGGATGCGACTGCTCAATGGCCAAC    CTGTGGACGCCAAGG
       CGATCGCCGCAG    ··············································    ···············    (-61bp)
```

Cas9 PAM-in

```
WT  :  TTGGCCGTGATGGGCAGTTCCGG       TGCCGGAAAGACGACCCTGCTGAATGCCCTTGCCTTTCGATCGCCGCAG    GGCATCCAAGTATCGCCATCCGGGATG

       TTGGCCGTGATGGGCAGTTCCGG       ·················································    ··CATCCAAGTATCGCCATCCGGGATG   (-51bp)
       TTGGC··················       ·················································    ···ATCCAAGTATCGCCATCCGGGATG   (-70bp) (X3)
```

(B) Cuts at a single target site

nD10A PAM-out

```
WT  :  CGATCGCCGCAG       GGCATCCAAGTATCGCCATCCGGGATGCGACTGCTCAATGGCCAAC      CTGTGGACGCCAA

       CGATCGCCGCAG       GGCATCCAAGTATCGCCATCCGGGATGCGACTGCTCAATGGCCAAC      ········GCCAA          (-8bp)
       CGATCGCCGCAG       ·········GTATCGCCATCCGGGATGCGACTGCTCAATGGCCAAC      CTGTGGACGCCAA          (-9bp)
       CGATCGCCGCAG       G······················ATGCGACTGCTCAATGGCCAAC       CTGTGGACGCCAA          (-23bp)
       CGATCGCCGCAG       MP                        ···················       ···TGGACGCCAA          (-22bp)
       CGATCGCCGCA        MP                        ···················       ···TGGACGCCAA          (-22bp)
       CGATCGCCGCAG       MP                    ·······················       CTGTGGACGCCAA          (-23bp)
       CGATCGCCGCAG       MP                       ····················       ··GTGGACGCCAA          (-23bp)
       CGATCGCCGCA        MP                    ·······················       ···TGGACGCCAA          (-26bp)
       CGATCGCCGCA        MP                ···························       ·TGTGGACGCCAA          (-27bp)
```

nH840A PAM-in

```
WT  :  TTGGCCGTGATGGGCAGTTCCGG       TGCCGGAAAGACGACCCTGCTGAATGCCCTTGCCTTTCGATCGCCGCAG    GGCATCCAAGTATCGCCATCCGGGATG

       TTGGCCGTGATGGGCAGTTCCGG       TGCCGGAAAGACGACCCTGCTGAATGCCCTTGCCTTTCGATCGCCGCAG    G· MP                         (-1bp)
       TTGGCCGTGATGGGCAGTTCCGG       ·····GAAAGACGACCCTGCTGAATGCCCTTGCCTTTCGATCGCCG       MP                            (-5bp)
       TTGGCCGTGATGGGCAGTTCCGG       MP                                               ·    ····TCCAAGTATCGCCATCCGGGATG   (-5bp)
       TTGGCCGTGATGGGCAGTTCCGG       TGCCGGAAAGACGACCCTGCTGAATGCCCTTGCCT MP     ·······    ·····CCAAGTATCGCCATCCGGGATG   (-12bp)
       TTGGCCGTGATGGGCAGTT           MP                            ·················AG    MP                            (-18bp) (x2)
       TTGGCCG·················      ·····G                                               MP                            (-21bp)
       TTGGCCGTGATGGGCAGTTC          MP                          ·····················    ···ATCCAAGTATCGCCATCCGGGATG   (-25bp)
```

Cas9 PAM-out

WT : CGATCGCCGCAG GGCATCCAAGTATCGCCATCCGGGATGCGACTGCTCAATGGCCAAC CTGTGGACGCCAAGG

CGATCGCCGCAG G·CATCCAAGTATCGCCATCCGGGATGCGACTGCTCAATGGCCAAC CTGTGGACGCCAAGG (-1bp)
CGATCGCCGCAG ··CATCCAAGTATCGCCATCCGGGATGCGACTGCTCAATGGCCAAC MP (-2bp) (X2)

(C) Simultaneous cuts at both target sites

nH840A PAM-in

WT : TTGGCCGTGATGGGCAGTTCCGG TGCCGGAAAGACGACCCTGCTGAATGCCCTTGCCTTTCGATCGCCGCAG GGCATCCAAGTATCGCCATCCGGGATG

TTGGCCGTGATGGGCAGTTCCGG ·············ACCCTGCTGAATGCCCTTGCCTTTCGATCGCCGCA· ····TCCAAGTATCGCCATCCGGGATG (-18bp)

Cas9 PAM-out

WT : CGATCGCCGCAG GGCATCCAAGTATCGCCATCCGGGATGCGACTGCTCAATGGCCAAC CTGTGGACGCCAAGG

CGATCGCCGCAG G·CATCCAAGTATCGCCATCCGGGATGCGACTGCTCAATGGCC··· ·TGTGGACGCCAAGG (-5bp)
CGATCGCCGCAG G···TCCAAGTATCGCCATCCGGGATGCGACTGCTCAATGGCCAA· ·TGTGGACGCCAAGG (-5bp)
CGATCGCC···· ·················ATCCGGGATGCGACTGCTCAATGGCCAA· ·TGTGGACGCCAAGG (-23bp)
CGATCGCCGCAG G····························ACTGCTCAATGGCCAAC ·TGTGGACGCCAAGG (-29bp)
CGATCGCCGCAG GG·····················GATGCGACTGCTCAATGGCCAA· ·············GG (-36bp)

Cas9 PAM-in

WT : TTGGCCGTGATGGGCAGTTCCGG TGCCGGAAAGACGACCCTGCTGAATGCCCTTGCCTTTCGATCGCCGCAG GGCATCCAAGTATCGCCATCCGGGATG

TTGGCCGTGATGGGCAGTTCCG· TGCCGGAAAGACGACCCTGCTGAATGCCCTTGCCTTTCGATCGCCGCAG G·CATCCAAGTATCGCCATCCGGGATG (-2bp) (x6)
TTGGCCGTGATGGGCAGTTCCGG ······AAAGACGACCCTGCTGAATGCCCTTGCCTTTCGATCGCCGCAG G·CATCCAAGTATCGCCATCCGGGATG (-7bp) (X4)
TTGGCCGTGATGGGCAGT····· ·GCCGGAAGACGACCCTGCTGAATGCCCTTGCCTTTCGATCGCCGCAG G·CATCCAAGTATCGCCATCCGGGATG (-7bp)
TTGGCCGTGATGGGCAGTTCCGG ······AAAGACGACCCTGCTGAATGCCCTTGCCTTTCGATCGCCGCAG G··ATCCAAGTATCGCCATCCGGGATG (-8bp) (X2)

(D) Large insertions (>200bp)

nD10A PAM-out

WT : CGATCGCCGCAG GGCATCCAAGTATCGCCATCCGGGATGCGACTGCTCAATGGCCAAC CTGTGGACGCCAAGG

CGATCGCCGCAG ACGTCATTTTCAACGCCATCCATGGTATGAGGGCTAATATCCCCGCCTGTGACGCGGGAGAAAAGGGGGGAAATG
CCCGCCTGGGAGCATCAGGAATTCTTTTTTATNTACTTTANNNNNGTTATATAAACAATTTTTGTTTTAATTGAATCTAATTTGCCATTGCTTTTAG
GAATCTCANGCATCCANCAAGCGTTTGTCCGCCGAATCGCCCNTCANTGAANAAGATCCTGTGGCG (+238bp) HDR (U6)

CGATCGCCGCAG ACGTCATTTTCAACGTCCTCGATAGTATAGTGGTTAGTATCCCCGCCTGTCACGCGGGAGACCGGGGTTCAATTC
CCGTCGGGGAGAATCTGTGATTCTTTTTTTTTTTCTTTTACTTTGTTATATAAACAATTTTTGTTTTAATTGAATCTAATTTGCCATTGCTTTTAGG
AATCTCAGGCATCCAGCAAGCGTTTGTCCGCCGAATCGCCCATCAGTGAAGAAGATCCTGTGGCGGCTACGAAAATCTCCCCGGCCATGTCGGCCTC
CACCTCCAGCGAAAAACCCATCAGCGAGCTGGCCACCTCTGTGCTGACCCACCGCTTTCCAGACTCCACCTCCTCACCCGGCGAACATGGCCTTGGA
CGAATGCAGTTGTCGATCCGCTACAGCGCCCAGCGTCAAAAACTAGACGTGACCATACACAAAATCCAGAAGATACCACTTCGCGATCCCAGCAA (+462bp) HDR (U6)

CGATCGCCGCGA ACGTCATTTTCAACGTCCTCGATAGTATAGTGGTTAGTATCCCCGCCTGTCACGCGGGAGACCGGGGTTCAAT
TCCCCGTCGGGGAGAATCTGTGATTCTTTTTTTTTTTCTTTTACTTTGTTATATAAACAATTTTTGTTTTAATTGAATCTAATTTGCCATTGCTTTT
AGGAATCTCAGGCATCCAGCAAGCGTTTGTCCGCCGAATCGCCCATCAGTGAAGAAGATCCTGTGGCGGCTACGAAAATCTCCCCGGCCATGTCGGC
CTCCACCTCCAGCGAAAAACCCATCAGCGAGCTGGCCACCTCTGTGCTGACCCACCGCTTTCCAGACTCCACCTCCTCACCCGGCGAACATGGCCTT
GGACGAATGCAGTTGTCGATCCGCTACAGCGCCCAGCGTCAAAAACTAGACGTGACCATACACAAAATCCAGAAGATACCACTTCGCGATCCCAGCA
ATATCCCCGATCCGTATGTTAAGCTGTATCTGTTGCCTGGACGCACCAAGGAGTCGAAACGCAAGACGAGCGTGATCAAGGACAACTGCAACCCCGT
CTACGATGCATCCTTTGAGTACCTGATTTCCATTGCCGAACTCAGGCAGACGGAACTGGAGGTGACGGTGTGCACCCAAAAGGGATTCCTATCCGGC
GGTAGTCCCATCATTGGCATGGTAGGTACCCGAAAGCAACCCCTTAGTTACAGACNCAGCGCGTACGTCCTTCGCATCCTTATGATTCCCAAGTACA
TATNTGCANANTACAGTATATATAGGAAAGANATCCNGNGAACTTCGNCGATACTTGGNTGCCCTGGTTTANAGCTA (+828bp) HDR (U6)

CGATCGCCGCAG MP· AGGCAATGGTCCATGGGTCAATGGGCGAAGGCCTTCAAGTCGATGGGGGTGA
CCNGGGTGGCCCCCTCNAACTTCCCCTCCGCCCGGGTCATANAATTGCCGTCGTCCTTGAAGAAGATGGTGCGCTCCTGGACGTAACCTTCGGGCAT
GGCGGACTTGAAAAAGTCGTGCTGCTTCATGTGGTCGGGGTAGCGGCTGAAGCACTGCACGCCGTANGNCAGGGTGGTCACCAAGGTGGGCCANGGC
ACGGGCAGCTTGCCGGTGGTGCANANGAACTTCANGGTCAGCTTGCCGTANGTGGCATCGCCCTCNCCCTCGCCGGACACGCTGAACTTGTGGGCGT
TTACNTCGCCGTCCANNTCGACCANGATGGGCACCACCCCGGTGAACANCTNCTCGCCCTTGNTCACCATGGTGGCGANCGGTGGATCCCGGGCCCG
CNGGTACCGTCNACTCTAGCGGNACCCCCNNNTGNNTNAGCTTGTTCAGCTGCGCTTGNTTNTTTGCNTAGCTTTCGCTTAGCGGANGTGNTCACTT
TGCTTGGTTTGAATTGGAATGTCGCTCCNTAGACNAAGCGCCTCTATTTATACTNCGGCNGTCGANGGTTCGAAATCGATNAGCTTGGANCTAATGA
ATAGCTCTAATGANT (-1bp) (+649) HDR (3xP3)

CGATCGCCGCAG GGCATCCAAGTATCGCCATCCGGGATGCGATGGCTCAATGGCCAA GGGCCAGGGCACGGGCAGCTT
GTTGCCGGTGGTGCAGATGAACTTCAGGGTCAGCTTGCCGTAGGTGGCATCGCCCTCGCCCTCGCCGGACACGCTGAACTTGTGGCCGTTTACGTC
GCCGTCCAGCTCGACCAGGATGGGCACCACCCCGGTGAACAGCTCCTCGCCCTTGCTCACCATGGTGGCGACCGGTGGATCCCGGGCCCGCGGTAC
CGTCGACTCTAGCGGTACCCCGATTGTTTAGCTTGTTCAGCTGCGCTTGTTTATTTGCTTAGCTTTCGCTTAGCGACGTGTTCACTTTGCTTGTTT
GAATTGAATTGTCGCTCCGTAGACGAAGCGCCTCTATTTATACTCCGGCGGTCGAGGGTTCGAAATCGATAAGCTTGGATCCTAATTGAATT
AGCTCTAATTGAATTAGTCTCTAATTGAATTAGATCCCCGGGCGAGCTCGCCTANG · CTGTGGACGCCAAGG (-1bp) (+452bp) HDR (3xP3)

Cas9 PAM-out

```
WT   :  CGATCGCCGCAG          GGCATCCAAGTATCGCCATCCGGGATGCGACTGCTCAATGGCCAAC      CTGTGGACGCCAAGG

CGATCGCCGCAG          G·CATCCAAGTATCGCCATCCGGGATGCGACTGCTCAATGG
ACTTGTGGCCGTTTACGTCGCCGTCCAGCTCGACCAGGATGGGCACCACCCCGGTGAACAGCTCCTCGCCCTTGCTCACCATGGTGGCGAC
CGGTGGATCCCGGGCCCGCGGTACCGTCGACTCTAGCGGTACCCCGATTGTTTAGCTTGTTCAGCTGCGCTTGTTTATTTGCTTAGCTTTC
GCTTAGCGACGTGTTCACTTTGCTTGTTTGAATTGAATTGTCGCTCCGTAGACGAAGCGCCTCTATTTATACTCCGGCGGTCGAGGGTTCG
AAATCGATAAGCTTGGATCCTAATTGAATTAGCTCTAATTGAATTAGTCTCTAATTGAATTAGATCCCCGGGCGAGCTCGCCTAGG
                                                              ·····           CTGTGGACGCCAAGG    (-6bp)(+359) HDR (3xP3)

CGATCGCCGCAG          TTTGACCATAGTGTTTCAATTCTACATTAATTTTACAGAGTAGAATGAAACGCCACCTACTCAGCCAA
GAGGCGAAAAGGTTAGCTCGCCAAGCAGAGAGGGCGCCAGTGCTCACTACTTTTTATAATTCTCAACTTCTTTTTCCAGACTCAGTTCGTA
TATATAGACCTATTTTCAATTTAACGTAACATCAAATTTTCTGTCAATAAAGCATATTTATTTATATTTATTTTACAGGAAAGAATTCCTT
TTAAAGTGTATTTTAACCTATAATGAAAAACGATTAAAAAAAAATACATAAAATAATTCGAAAATTTTTGAATAGCCCAGGTTGATAAAAA
TTCATTTCATACGTTTTATAACTTATGCCCNTAAGTATTT          CGACTGCTCAATGGCCAAC      CTGTGGACGCCAAGG    (+382bp) HDR (U6)

CGATCGCCGCAG          CAACTGCAACCCCGTNTACGATGCATCCTTTGAGTACNTGATTTCCATTGCCGAACTCAGGCAGACGG
AACTGGAGGTGACGGTGTGCACCCAAAAGGGATTCCTATCCGGCGGTAGTCCCATCATTGGCATGGTAGGTACCCGAAAGCAACCCCTTAG
TTACAGACACAGCGCGTACGTCCTTCGCATCCTTATGATTCCCAAGTACATATTCTGCAAGAGTACAGTATATATAGGAAAGATATCCGGG
TGAACTTCGGCGATACTTGGATGCCCTGGTTTTAGAGCTAGAAATAGCAAGTTAAAATAAGGCTAGTCCGTTATCAACTTGAAAAAGTGGC
ACCGAGTCGGTGCTTTTTTGCTCACCTGTGATTGCTCCTACTCAAATACAAAAACATCAAATTTTCTGTCAATAAAGCATATTTATTTATA
TTTATTTTACAGGAAAGAATTCCTTTTAAAGTGTATTTTAACCTATAATGAAAAACGATTAAAAAAAAATACATAAAATAATTCGAAAATTT
TTGAATAGCCCAGGTTGATAAAAATTCATTTCATACGTTTTATAACTTATGCCCCTAAGTATTTTTTGACCATAGTGTTTCAATTCTACAT
TAATTTTACAGAGTAGAATGAAACGCCACCTACTCAGCCAAGAGGCGAAAAGGTTAGCTCGCCAAGCAGAGAGGGCGCCAGTGCTCACTAC
TTTTTATAATTCTCAACTTCTTTTTCCAGACTCAGTTCGTATATATAGACCTATTTTCAATTTAACGT
                                                  CGACTGCTCAATGGCCAAC      CTGTGGACGCCAAGG     (+773bp) HDR (U6)
```

(E) Indels

nD10A PAM-out

```
WT  :  CGATCGCCGCAG        GGCATCCAAGTATCGCCATCCGGGATGCGACTGCTCAATGGCCAAC      CTGTGGACGCCAAGG

       CGATCGCCGCAG        G··············································      ···TGGACGCCAAGG          (-49bp) (+3bp)
                            AGA
       CGATCGCCGCA·        ···············································      ··GTGGACGCCAAGG          (-49bp) (+3bp)
                 ATC
       CGATCGCCGCAG        GGCATCCAAGTATCGCCATCCGGGATGCGATTGTTGT·TGG···AC      ·TGTGGACGCCAAGG          (-5bp)  (+4bp) (S 4bp)
                                                       GAAC
       CGATCGCCG··    ·    ·GCAT···········TTCCGGGATGCGATGGCTCAATGGCCAAC      CTGTGGACGCCAAGG          (-15bp) (+3bp) (S 1bp)
                                                    CCC
```

nH840A-PAM-in

```
WT  :  TTGGCCGTGATGGGCAGTTCCGG      TGCCGGAAAGACGACCCTGCTGAATGCCCTTGCCTTTCGATCGCCGCAG    GGCATCCAAGTATCGCCATCCGGGATG

       TTGGCCGTGATGGGCAGTTCCGG      MP                                             A···    ·······AAGTATCGCCATCCGGGATG   (-10bp) (+1bp)
       TTGGCCGTGATGGGCAGTTCCGG      TTGCCGGAAAGACGACCCTGCTGAATGCCCTTGCCTTTCGATCGCCGCAG    G·········· MP                (-10bp) (+1bp)
       TTGGCCGT···············      · ···············································    GGCATCCAAGTATCGCCATCCGGGATG   (-64bp) (+10bp)  (X2)
               CCAAACTTTT
       TTGGCCGTGATGGGCAGTTC         MP                GGCAACTGGGTTGGGAA················    ···················CGGGATG   (-59bp) (+16bp)
```

Cas9-PAM-out

```
WT  :  CGATCGCCGCAG        GGCATCCAAGTATCGCCATCCGGGATGCGACTGCTCAATGGCCAAC      CTGTGGACGCCAAGG

       CGATCGCCGCAG        GGCATCCAAGTATCGCCATCCGGGATGCGACTGCTCAATGGCCA··      CTGTGGACGCCAAGG             (-2bp)  (+2bp)  (X2)
                                                                     TG
       CGATCGCCGCA·        ····TCCAAGTATCGCCATCCGGGATGCGACTGCTCAATGGC·A··      ··GTGGACGCCAAGG             (-8bp)  (+1bp)
                                                                 G
       CGATCGCCGCAG        GGCATCCAAGTATCGCCATCCGGGATGCGACTGCTCA·········      CTGTGGACGCCAAGG             (-9bp)  (+1bp)
                             G
       CGATCGCCGCA· TCGAT  ·············GCCATCCGGGATGCGACTGCTCAATGGC·A··      ··GTGGACGCCAAGG             (-14bp) (+5bp)
       CGATCGCCGCAG CTGCGATCG ············································      CTGTGGACGCCAAGG             (-47bp) (+9bp)
       CGATCGCCGC·· GGCCAC ···············································      ··GTGGACGCCAAGG             (-50bp) (+6bp)
       CGATCGCCGCAG        GG····················GATGCGACTGCTCAATGGACT·G      CTGTGGACGCCAAGG             (-23bp) (S 3bp)
```

Cas9-PAM-in

```
WT  :  TTGGCCGTGATGGGCAGTTCCGG       TGCCGGAAAGACGACCCTGCTGAATGCCCTTGCCTTTCGATCGCCGCAG    GGCATCCAAGTATCGCCATCCGGGATG

       TTGGCCGTGATGGGCAGTTCCGGGAT    .................................................    GGCATCCAAGTATCGCCATCCGGGATG   (-49bp) (+3bp)
```

Fig S2 - Related to Fig.2 - Variety of resistant alleles produced by the regular Cas9 and nickase versions combined with our CopyCat elements. a-e. These sequences were recovered by sequencing F_2 males from crosses showing super-Mendelian inheritance rates in Fig. 2. The *w2*, *w8* and *w9* gRNA sequences are indicated in blue, orange and green, respectively. PAM sequences are highlighted in red. Dots represent deletions observed in each genotype. Nucleotide insertions were highlighted in pink while substitutions appeared underlined. The different resistant allele categories identified in each genotype are shown. **a.** Large deletions represent mutations erasing more than 50bp and spanning both cut sites. **b.** Resistant alleles contain single mutations occurring at one cut site but not at the other. In all cases, these mutations were smaller than 50 nucleotides. Also, our Sanger sequencing analysis displayed messy traces where the exact sequence could not be determined. These alterations showed multiple peaks (MP) in our Sanger sequencing analysis and suggest that the gRNA could cut after germline formation, causing genetic mosaicism. These events are represented by an MP when needed. **c.** Simultaneous resistant alleles were also formed at both target sites. All these deletions were smaller than 50 base pairs. **d.** Large insertions bigger than 200 nucleotides representing partial HDR events were also found. **e.** A combination of deletions and small insertions together in the same animal were also detected. To keep an appropriate sequence alignment in this category, some insertions were allocated under the main alignment sequence line, right after the nucleotide where it was inserted.
